# Properties of non-structural protein 1 of Semliki Forest virus and its interference with virus replication

**DOI:** 10.1099/vir.0.2008/000299-0

**Published:** 2008-06

**Authors:** Kaja Kiiver, Ingrid Tagen, Eva Žusinaite, Nele Tamberg, John K. Fazakerley, Andres Merits

**Affiliations:** 1 Institute of Technology, University of Tartu, Tartu, Estonia; 2 Estonian Biocentre, Tartu, Estonia; 3 Centre for Infectious Diseases, College of Medicine and Veterinary Medicine, University of Edinburgh, Edinburgh, UK

## Abstract

Semliki Forest virus (SFV) non-structural protein 1 (nsP1) is a major component of the virus replicase complex. It has previously been studied in cells infected with virus or using transient or stable expression systems. To extend these studies, tetracycline-inducible stable cell lines expressing SFV nsP1 or its palmitoylation-negative mutant (nsP1^6D^) were constructed. The levels of protein expression and the subcellular localization of nsP1 in induced cells were similar to those in virus-infected cells. The nsP1 expressed by stable, inducible cell lines or by SFV-infected HEK293 T-REx cells was a stable protein with a half-life of approximately 5 h. In contrast to SFV infection, induction of nsP1 expression had no detectable effect on cellular transcription, translation or viability. Induction of expression of nsP1 or nsP1^6D^ interfered with multiplication of SFV, typically resulting in a 5–10-fold reduction in virus yields. This reduction was not due to a decrease in the number of infected cells, indicating that nsP1 expression does not block virus entry or initiation of replication. Expression of nsP1 interfered with virus genomic RNA synthesis and delayed accumulation of viral subgenomic RNA translation products. Expression of nsP1 with a mutation in the palmitoylation site reduced synthesis of genomic and subgenomic RNAs and their products of translation, and this effect did not resolve with time. These results are in agreement with data published previously, suggesting a role for nsP1 in genomic RNA synthesis.

## INTRODUCTION

*Semliki Forest virus* (SFV) belongs to the genus *Alphavirus* (family *Togaviridae*). Along with Sindbis virus (SV), the prototype member of the genus, SFV is one of the most studied alphaviruses. The genome of SFV is a positive-strand RNA of approximately 11.5 kb with a 5′ cap and a 3′ terminal poly(A) tract (Kääriäinen *et al.*, 1987). The 5′ two-thirds of the genome encodes the non-structural (ns) polyprotein P1234. In infected cells, P1234 is autocatalytically cleaved into four individual non-structural proteins designated nsP1, nsP2, nsP3 and nsP4 (Kääriäinen *et al.*, 1987). The SFV-encoded protease, nsP2, is responsible for proteolytic processing of P1234 (Merits *et al.*, 2001; Lulla *et al.*, 2006a). The first cleavage takes place at the nsP3/4 cleavage site and releases mature nsP4, which together with the remaining P123 polyprotein forms the early minus-strand RNA replicase. In contrast, the late replicase complex consists of fully processed non-structural proteins and synthesizes positive-sense genomic and subgenomic (sg) RNA molecules (Lemm *et al.*, 1994; Shirako & Strauss, 1994; Vasiljeva *et al.*, 2003; Kim *et al.*, 2004).

nsP1 participates directly in initiation and elongation of the minus-strand RNA synthesis of alphaviruses (Sawicki *et al.*, 1981; Hahn *et al.*, 1989; Wang *et al.*, 1991). During minus-strand RNA synthesis, nsP1 interacts with nsP4 (Shirako *et al.*, 2000; Fata *et al.*, 2002). The properties of nsP1 of SFV and SV have been studied previously using infected cells, transient and stable expression systems, and purified recombinant proteins. The N-terminal region of nsP1 is a methyltransferase and guanylyltransferase involved in capping the viral positive-strand RNAs (Mi *et al.*, 1989; Mi & Stollar, 1991; Laakkonen *et al.*, 1994; Ahola & Kääriäinen, 1995; Wang *et al.*, 1996; Ahola *et al.*, 1997). nsP1 is also associated with cellular membranes (Peränen *et al.*, 1995) and serves as the sole membrane anchor for the virus replicase complex (Salonen *et al.*, 2003). Two different events are involved in binding of nsP1 to cellular membranes. Firstly, the amphipathic peptide segment, located in the central part of nsP1, binds to cellular membranes (Ahola *et al.*, 1999; Lampio *et al.*, 2000). This binding is required for activation of the enzymic activities of nsP1 and for infectivity of the viral genome (Spuul *et al.*, 2007). Secondly, post-translational palmitoylation at cysteine residues 418–420 is responsible for strong membrane association of the protein (Laakkonen *et al.*, 1996). Palmitoylation as such is not required for the enzymic activities of nsP1 or for infectivity of the viral genome (Ahola *et al.*, 2000; Žusinaite *et al.*, 2007). However, the deletion or substitution of cysteines 418–420 in nsP1 of SFV results in a severe defect in virus replication and causes accumulation of compensatory mutations. This effect may result from the incorrect subcellular localization of mutant nsP1 and/or from its inability to interact with nsP4 (Žusinaite *et al.*, 2007). Li *et al.* (1997) demonstrated that stable transfectants of Hela cells, expressing nsP1 from low-methionine (LM)-adapted SV, SV(LM21), were able to support replication of standard SV in cells maintained in LM medium. The expression of full-length nsP1 had only a minor affect on replication of SV(LM21), but truncated forms of nsP1 acted as dominant-negative mutants.

Within the first few hours, alphavirus infection results in exclusion of superinfection by homologous virus (Adams & Brown, 1985) and, in the case of vertebrate cells, in shutdown of host-cell gene expression and induction of apoptotic death. It has been shown or proposed that free nsP2 is required for these effects (Karpf *et al.*, 1997; Garmashova *et al.*, 2006, 2007; Sawicki *et al.*, 2006); however, it has not been demonstrated whether expression of other non-structural proteins causes similar effects. nsP1 expression in infected or transfected cells results in prominent changes, such as induction of filopodia-like structures and rearrangements of actin filaments (Laakkonen *et al.*, 1998). These nsP1-induced effects make it another candidate for superinfection exclusion and/or virus-induced cytotoxic effects.

We studied the properties of nsP1 and its effect on host cells and on SFV infection by combining two powerful approaches, tetracycline-inducible stable cell lines and recombinant SFV genomes carrying marker genes in the replicase or the structural open reading frame (ORF). nsP1 expressed alone in the absence of the other virus replicase proteins was a stable protein with a half-life of about 5 h; stability was not affected by virus infection. Expression of nsP1 induced prominent changes in cell membranes and cell morphology, but had no significant effect on cell viability, virus entry or establishment of virus replication. However, nsP1 expression suppressed genomic RNA synthesis leading to reduced virus yield. The palmitoylation-negative form of nsP1 acted as a dominant-negative mutant and suppressed both genomic and sgRNA synthesis.

## METHODS

### Plasmids and construction of inducible cell lines.

pcDNA4/TO (Invitrogen) plasmids encoding SFV nsP1 and nsP1^6D^ (harbouring the mutation ^418^CCC^420^→AAA in the palmitoylation site) were kindly provided by Professor Leevi Kääriäinen (Institute of Biotechnology, University of Helsinki, Finland). Each plasmid (10 μg) was linearized by *Pvu*I and electroporated into HEK293 T-REx cells, and zeocin (30 μg ml^−1^) was added to select transfectants. The expression of recombinant protein was induced by adding tetracycline (1 μg ml^−1^) and verified by Western blotting at 12 h post-induction; the cell lines expressing inducible nsP1 and nsP1^6D^ were designated T-REx-nsP1 and T-REx-nsP1^6D^, respectively, and were grown at 37 °C with 5 % CO_2_ in Iscove's modified Dulbecco's medium (IMDM; Gibco) supplemented with 10 % fetal calf serum, blasticidin (5 μg ml^−1^) and zeocin (30 μg ml^−1^).

### SFV, recombinant viruses and cell infection.

BHK-21 cells, used for multiplication and plaque titration of SFV stocks, were grown as described previously (Žusinaite *et al.*, 2007). SFV4(3H)-Rluc and SFV4-StRluc containing insertion of the *Renilla* luciferase (Rluc) reporter were constructed as described by Tamberg *et al.* (2007) and Thomas *et al.* (2003), respectively. SFV4, SFV4(3H)-Rluc and SFV4-StRluc were generated from corresponding infectious cDNAs, as described previously (Liljeström & Garoff, 1991), grown and plaque titrated in BHK-21 cells, and used for infection of HEK293 T-REx cells or constructed cell lines.

### Analysis of transcription and translation in stable cell lines.

T-REx-nsP1 cells were grown on 60 mm dishes to 75 % confluency and induced with tetracycline for 24 h; uninduced cultures were used as controls. RNA transcripts were labelled with 10 μCi (370 kBq) [5-^3^H]uridine (Amersham Biosciences) in 1 ml serum-free IMDM for 1 h. Cells were then collected, washed with PBS and lysed in 200 μl 1 % SDS, followed by heating at 65 °C for 5 min. To label cellular proteins, the induced cells were first incubated for 30 min in methionine- and cysteine-free medium, followed by pulse labelling with 50 μCi (1.85 MBq) [^35^S]methionine and [^35^S]cysteine for 30 min. Pulse–chase experiments were carried out as described by Lulla *et al.* (2006b). The lysates of 5-^3^H- or ^35^S-labelled cells were precipitated with 1 ml 10 % trichloroacetic acid and the incorporated radioactivity was measured using a 1414 Liquid Scintillation Counter (Wallac).

### Analysis of transcription and translation in SFV4-infected cells.

HEK293 T-REx cells (1×10^6^) were infected with SFV4 at an m.o.i. of 20 for 24 h and labelled with either 10 μCi [5-^3^H]uridine or 50 μCi [^35^S]methionine and [^35^S]cysteine as described above. For the transcription analysis, total RNA from equal amounts of infected and mock-infected cells was isolated using Trizol reagent (Invitrogen) and dissolved in 30 μl DEPC-treated water. Total RNA (10 μl) was subjected to denaturing formaldehyde agarose gel electrophoresis (Sambrook & Russell, 2001). The RNA bands corresponding to cellular pre-mRNAs and mRNAs (the gel area above the SFV4 42S RNA band) were excised from the gel, placed into 3 ml scintillation liquid and the radioactivity quantified by liquid scintillation counting. The translation analysis was carried out as described by Tamm *et al.* (2008). Inhibition of cellular translation was assessed by measuring the radioactivity incorporated into the protein band corresponding to actin, as described previously (Gorchakov *et al.*, 2004). Immunoprecipitation of [^35^S]methionine- and [^35^S]cysteine-labelled samples was carried out as described previously (Tamm *et al.*, 2008).

### Immunofluorescence and confocal microscopy.

Cells at 75 % confluency were induced with tetracycline or infected with SFV4 at an m.o.i. of 20 for 6 h. Uninduced or mock-infected cells were used as a control. Cells were then fixed, permeabilized and treated with specific anti-nsP1 antibodies (Laakkonen *et al.*, 1994) and rhodamine-conjugated concanavalin A (ConA; Sigma) for plasma membrane staining as described previously (Spuul *et al.*, 2007) and analysed with a Bio-Rad MRC-1024 confocal microscope.

### Immunoblotting.

Lysates prepared from equal amounts of induced or uninduced cells were separated by SDS-PAGE (∼10^5^ cells per cell line). Proteins were transferred to a membrane and detected by antibodies against nsP1 using enhanced chemiluminescence.

### WST-1 assay for cell viability.

T-REx-nsP1 and T-REx-nsP1^6D^ cells were seeded in 96-well plates (9×10^4^ cells per well) and allowed to grow for 18 h. Recombinant protein expression was induced for 24 h; control cells remained uninduced. For comparison, the parental HEK293 T-REx cell line was infected with SFV4 at an m.o.i. of 20 or was mock infected, and cells were incubated for 24 h. Subsequently, 20 μl WST-1 (Roche) was added to each well and the plate was incubated for a further 1 h under the cell culture growing conditions. *A*_450_ was read in a microplate reader. This experiment was repeated at least three times for each cell line.

### Analysis of Rluc activity in infected cells.

HEK293 T-REx, T-REx-nsP1 and T-REx-nsP1^6D^ cells grown on 35 mm dishes were induced with tetracycline or mock induced, and infected with SFV4(3H)-Rluc or SFV4-StRluc at an m.o.i. of 0.6. EnduRen Live Cell Substrate (Promega) was added to the medium immediately after infection and the Rluc activity was measured at selected time points using a Glomax 20/20 luminometer (Promega).

### Northern blot analysis.

HEK293 T-REx, T-REx-nsP1 and T-REx-nsP1^6D^ cells grown on 35 mm dishes were induced with tetracycline or mock induced, and infected with SFV4(H3)-Rluc at an m.o.i. of 0.6. At 12 h post-infection (p.i.), total RNA from infected cells was purified using Trizol reagent (Invitrogen). Each RNA sample (10 μg) was used for Northern blotting as described previously (Tamm *et al.*, 2008) using an RNA probe complementary to the positive-strand of SFV genomic (nt 10951–11445) and sgRNA.

### Statistical methods and software.

The data from radioactive gels were quantified using Image Quant (Molecular Dynamics) software. Student's *t*-test was used for calculation of statistical significance.

## RESULTS

### Evaluation of the half-life of nsP1 and its subcellular localization

**Fig. 1. f1:**
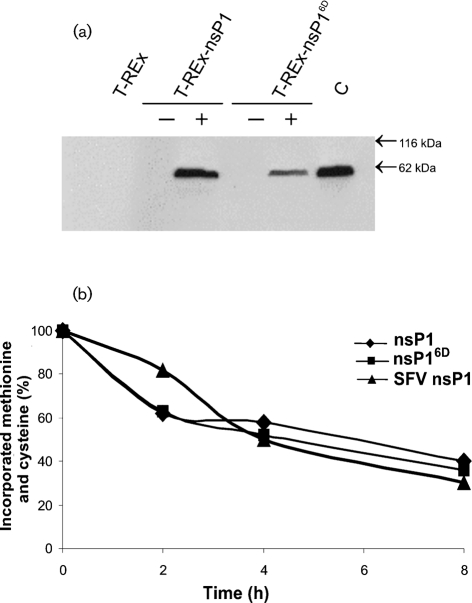
Expression of SFV nsP1 and nsP1^6D^ by inducible cell lines (a) and determination of the half-life of SFV nsP1 and nsP1^6D^ (b). (a) Western blot analysis of recombinant protein expression in T-REx-nsP1 and T-REx-nsP1^6D^ cell lines. +, Tetracycline-induced cells; –, mock-induced controls. Lane C represents a lysate from SFV-infected HEK293 T-REx cells. (b) nsP1 or nsP1^6D^ from pulse-chased cells was immunoprecipitated, separated by SDS-PAGE and analysed by phosphorimaging. Each experiment was performed at least twice, with no significant difference between the datasets (Student's *t*-test). The amount of incorporated label immediately after the pulse (0 h) (inducible cell lines) or after a 1 h chase (for SFV-infected cells; as in infected cells, the amount of nsP1 initially increases as a result of its release from precursors) was taken as 100 %. Quantification was made for label incorporated into immunoprecipitated nsP1 from induced cell lines (⧫) and from infected cells (▴) and for label incorporated into immunoprecipitated nsP1^6D^ from induced cell lines (▪).

Stably transfected cell lines inducible for the expression of wild-type (wt) nsP1 or its palmitoylation-negative form (nsP1^6D^) were constructed and analysed. Tetracycline-induced expression of nsP1 and nsP1^6D^ was confirmed by immunoblotting (Fig. 1a[Fig f1]). The level of wt nsP1 expression reached a plateau at 12 h post-induction (data not shown) and was similar to that of SFV4-infected HEK293 T-REx cells at 6 h p.i. The expression of nsP1^6D^ was somewhat lower than that of wt nsP1. Thus, in contrast to previously reported Hela cell lines expressing SV nsP1 (Li *et al.*, 1997), induced T-REx-nsP1 cells did not overexpress SFV nsP1. In our stably transfected inducible T-REx cells, the half-life of nsP1 and nsP1^6D^, as measured by metabolic labelling, was 5 h (Fig. 1b[Fig f1]), indicating that nsP1 was highly stable and that this was not changed by mutation of the palmitoylation site. For comparison, the half-life of nsP1 in SFV4-infected HEK293 T-REx cells was determined and found to be approximately 4 h (Fig. 1b[Fig f1]).

**Fig. 2. f2:**
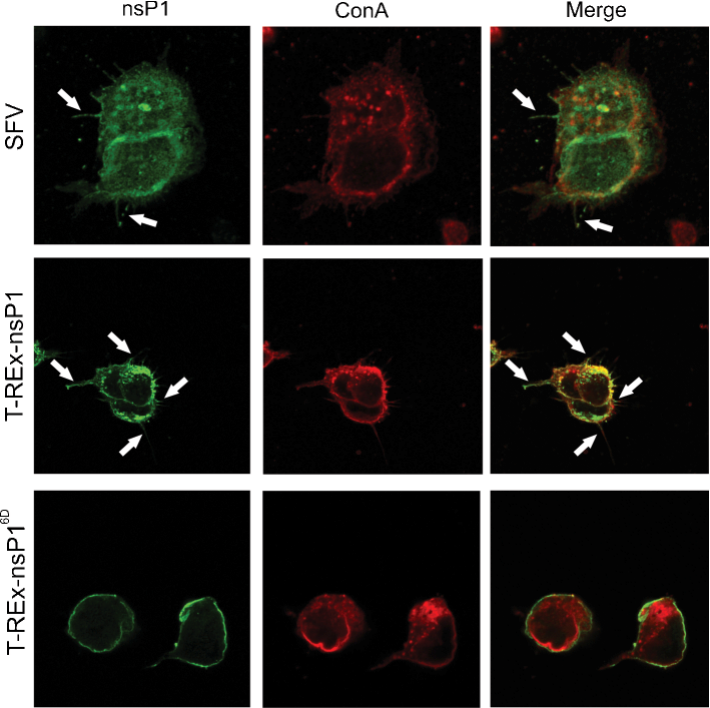
Subcellular localization of the SFV nsP1 and nsP1^6D^. T-REx cells were induced with tetracycline (1 μg ml^−1^) for 24 h or infected with SFV4 at an m.o.i. of 20 for 6 h. The cells were then double-stained with nsP1-specific rabbit polyclonal antibody and rhodamine-conjugated ConA. The arrows indicate filopodia-like structures.

In induced T-REx-nsP1 cells, nsP1 localized mostly to the plasma membrane, resulting in the extensive formation of filopodia-like structures. In contrast to SFV4-infected HEK293 T-REx cells, induced nsP1 did not localize to intracellular vesicles (Fig. 2). nsP1^6D^ also localized predominantly to the plasma membrane, but few filopodia-like structures were induced (Fig. 2). Thus, the half-life and, to some extent, the subcellular localization of nsP1 in induced cell lines were similar to those observed in infected cells.

### Effects of nsP1 expression

**Fig. 3. f3:**
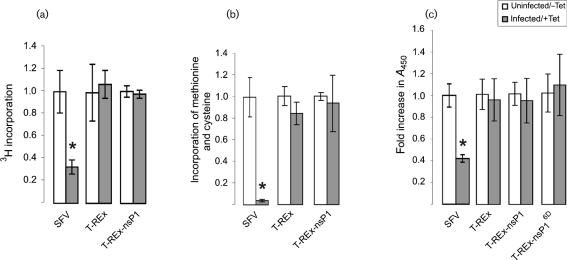
Analysis of transcription, translation and cell viability. (a, b) Cells were induced with tetracycline (shaded columns) or mock induced (open columns). After 24 h, cells were labelled for 1 h with 10 μCi [5-^3^H]uridine (a) or for 1 h with 50 μCi [^35^S]methionine/cysteine (b). The mean level of ^3^H or ^35^S incorporation in each uninduced cell line was taken as 1 for the corresponding induced cell line. (c) Cells were induced (shaded columns) with tetracycline for 24 h or mock induced (open columns). To measure cell viability, 1 h after adding the WST-1 reagent, *A*_450_ was measured. The absorbance values for each tetracycline-induced cell line were normalized to the mean value of the same cell line without tetracycline. The values for virus-infected cells were normalized to uninfected cells. T-REx indicates the parental HEK293 T-REx cell line and SFV indicates HEK293 T-REx cells infected with SFV4 at an m.o.i. of 20 for 24 h. Each column represents the mean±sd from three experiments. An asterisk indicates a statistically significant difference (*P*<0.05; Student's *t*-test) between experimental and control cells.

The effect of nsP1 expression on cellular transcription and translation was analysed by metabolic labelling. As expected, SFV4 infection significantly reduced synthesis of cellular pre-mRNA and mRNA. The reduction was approximately threefold (Fig. 3a[Fig f3]). In contrast, there was no statistically significant reduction in cellular transcription following induction of nsP1 expression (Fig. 3a[Fig f3]). Similarly, SFV4 infection led to an almost complete shutdown of cellular translation by 24 h p.i. (Fig. 3b[Fig f3]), whilst induction of nsP1 expression caused no significant effect.

As nsP1 localizes on the plasma membrane, it may act as an inducer of cell death, for example by activating cell-surface deaths receptors. A WST-1 cell viability assay revealed that, whilst SFV4 infection considerably reduced viability of HEK293 T-REx cells at 24 h p.i., induction of nsP1 or nsP1^6D^ expression had no effect on cell viability (Fig. 3c[Fig f3]). Taken together, these results showed that SFV nsP1 expression causes significant morphological changes in cells but on its own has no effect on transcription, translation or viability.

### nsP1 expression by HEK293 cells does not affect virus entry but interferes with virus replication

**Fig. 4. f4:**
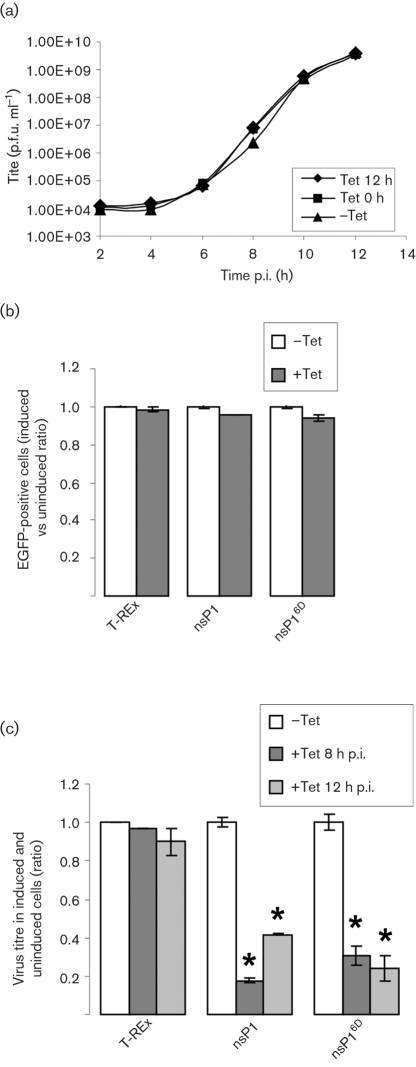
Effect of tetracycline treatment on the replication of SFV4 in HEK293 T-REx cells (a) and the effects of tetracycline-induced expression of nsP1 or nsP1^6D^ on the extent of infection with SFV VLPs (b) or production of infectious SFV4 (c). (a) HEK293 T-REx cells (10^6^) were treated with tetracycline 12 h before infection (⧫), at the same time as infection (▪) or were mock treated (▴). Cells were infected with SFV4 at an m.o.i. of 0.2, and the samples were collected at the indicated time points and titrated by a standard plaque assay on BHK-21 cells. The experiment was repeated twice with no significant difference between the datasets (Student's *t*-test). (b) HEK293 T-Rex, T-REx-nsP1 and T-REx-nsP1^6D^ cells (10^6^) induced with tetracycline for 12 h (shaded columns) or mock induced (open columns) were infected with SFV1–d1EGFP1 VLPs at an m.o.i. of 5. The percentage of d1EGFP-positive cells (determined at 12 h p.i. using a BD LSR II cell sorter) in mock-induced samples was taken as 1 for the corresponding induced cell line. (c) HEK293 T-Rex, T-REx-nsP1 and T-REx-nsP1^6D^ cells (10^6^) induced with tetracycline for 12 h or mock induced were infected with SFV4 at an m.o.i. of 0.6. The samples were collected at 8 and 12 h p.i. and titrated. The titre of the virus stock obtained from the mock-induced cells (open columns) was taken as 1 for the corresponding stock from induced cells. Dark-shaded columns represent the relative titre of the virus stock from induced cells at 8 h p.i. and light-shaded columns represent the relative titre of the virus stock from induced cells at 12 h p.i. All experiments were repeated at least twice with closely similar results. (b, c) Each column represents the data of three experiments (means±sd). An asterisk indicates a statistically significant difference (*P*<0.05; Student's *t*-test).

The growth curves of SFV4 in HEK293 T-REx cells, treated or mock treated with tetracycline 12 h prior to infection or at the same time as infection, were essentially identical (Fig. 4a[Fig f4]). The same result was obtained for infection of these cells by SFV4 viruses carrying a *Renilla* luciferase marker gene in the non-structural ORF [SFV4(3H)-Rluc] or in the structural ORF (SFV4-StRluc) (data not shown). As the addition of tetracycline did not have any effect on the replication of SFV in HEK293 T-REx cells and 12 h treatment achieved expression of recombinant protein at levels typically found in SFV-infected cells, these conditions were used in subsequent experiments.

Recombinant nsP1 and nsP1^6D^ localized to the plasma membrane of induced cells. To investigate whether this had any effect on the entry of SFV, the T-REx-nsP1, T-REx-nsP1^6D^ and HEK293 T-REx cells were infected with virus-like particles (VLPs) containing packaged SFV1–d1EGFP replicons, capable of infection and d1EGFP expression, but unable to produce infectious progeny (Žusinaite *et al.*, 2007). The percentage of d1EGFP-positive cells at 12 h p.i. for HEK293 T-REx and uninduced T-REx-nsP1 cells was found to be approximately five times lower than for BHK-21 cells. The susceptibility of uninduced T-REx-nsP1^6D^ cells was reduced even further, reaching only 25 % that of HEK293 T-REx cells. These relative susceptibilities were reproduced when the full-length EGFP-expressing virus SFV4(3H)–EGFP (Tamberg *et al.*, 2007) was used for infection of these cell lines. Therefore, in all subsequent experiments the number of virions or VLPs used for infection of each cell line was selected according to the individual susceptibilities of the cell lines, e.g. four times more infectious material was used for infection of T-REx-nsP1^6D^ cells to achieve the same m.o.i. as for HEK293 T-REx or T-REx-nsP1 cells. Induction of nsP1 or nsP1^6D^ expression did not affect infection efficiency in any cell line (Fig. 4b[Fig f4]). Thus, expression of nsP1 or nsP1^6D^ did not affect the ability of SFV VLPs to enter into cells and establish replication.

To determine whether expression of nsP1 or nsP1^6D^ interferes with SFV infection, induced or mock-induced HEK293 T-REx, T-REx-nsP1 and T-REx-nsP1^6D^ cell lines were infected with SFV4 and the virus titres at 8 and 12 h p.i. were compared. As expected, addition of tetracycline 12 h prior to infection did not have a significant effect on the multiplication of SFV4 in HEK293 T-REx cells (Fig. 4c[Fig f4]). In contrast, induction of the expression of nsP1 or nsP1^6D^ significantly decreased SFV4 titres (Fig. 4c[Fig f4]). At 8 h p.i., the decrease in titre was greater in induced T-REx-nsP1 cells than in induced T-REx-nsP1^6D^ cells. At 12 h p.i., the situation was reversed: the titre from induced T-REx-nsP1 cells was only decreased 2.5-fold compared with almost a 5-fold decrease observed in induced T-REx-nsP1^6D^ cells (Fig. 4c[Fig f4]). Thus, the expression of wt nsP1 of SFV or its palmitoylation-negative mutant inhibited extracellular accumulation of virus.

### Expression of nsP1 or nsP1^6D^ interferes with different steps in the SFV infection process

**Fig. 5. f5:**
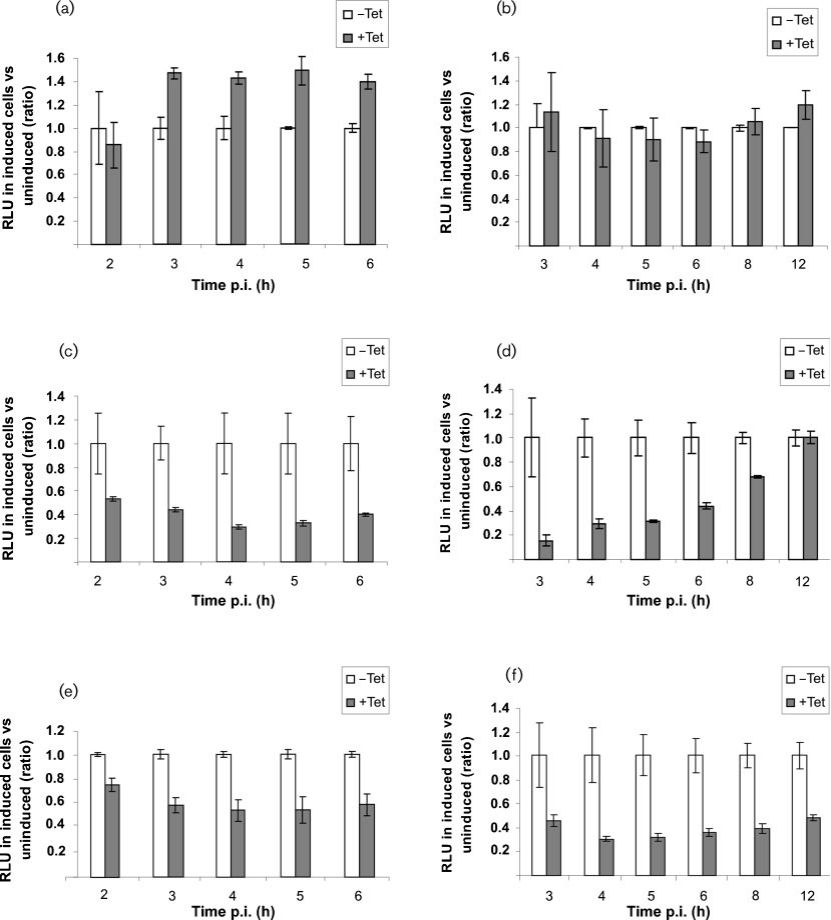
Effect of induction of nsP1 or nsP1^6D^ on the expression of a marker protein from the virus replicase of the structural ORF. HEK293 T-Rex (a, b), T-REx-nsP1 (c, d) and T-REx-nsP1^6D^ (e, f) cells (10^6^), induced with tetracycline for 12 h (shaded columns) or mock induced (open columns), were infected with SFV4(3H)-Rluc (a, c, e) or SFV4-StRluc (b, d, f) at an m.o.i. of 0.6. EnduRen Live Cell Substrate (Promega) was added to the medium immediately after infection and Rluc activity was measured at selected time points. At each time point, the Rluc activity in mock-induced samples was taken as 1 for the corresponding induced samples. Each column represents the data of three parallel samples (means±sd). The experiment was repeated twice with closely similar results.

Given that nsP1 did not affect virus entry (Fig. 4b[Fig f4]), the reduced extracellular virus titres observed following nsP1 or nsP1^6D^ expression could reflect defects in production of viral transcripts, virus structural proteins, or assembly and release of virions. To determine whether expression of nsP1 or nsP1^6D^ had any effect on virus protein synthesis, the reporter viruses SFV4(3H)-Rluc and SFV4-StRluc were used to monitor the translation of genomic and sgRNAs. Translation of genomic RNA is proportional to its copy number only at early stages of infection; late in infection, translation of genomic RNAs is inhibited but RNA synthesis remains active. Expression of RLuc in cells infected with SFV4(3H)-Rluc was therefore measured only during the first 6 h of infection. Rluc activity produced by SFV4-StRluc should be proportional to the amount of expressed viral structural proteins throughout the course of infection and this was monitored up to 12 h p.i. Treatment of HEK293 T-REx cells with tetracycline prior to infection with marker viruses resulted in increased translation of the replicase ORF (Fig. 5a[Fig f5]) but had no effect on translation of the structural protein ORF (Fig. 5b[Fig f5]). In contrast, tetracycline induction of the T-REx-nsP1 cell line resulted in up to a 4-fold reduction in replicase ORF-derived Rluc activity (Fig. 5c[Fig f5]). Marker expression, mediated by the sgRNA, was strongly suppressed only in the early stages of infection; by 12 h p.i., no difference between induced and uninduced cells was detectable (Fig. 5d[Fig f5]). Induction of the T-REx-nsP1^6D^ cell line resulted in less than 2-fold reduction in replicase ORF-derived Rluc activity (Fig. 5e[Fig f5]), but caused strong and persistent inhibition of structural protein ORF-derived RLuc activity (Fig. 5f[Fig f5]), indicating that the mechanism of action of the mutant protein differed from that of wt nsP1.

**Fig. 6. f6:**
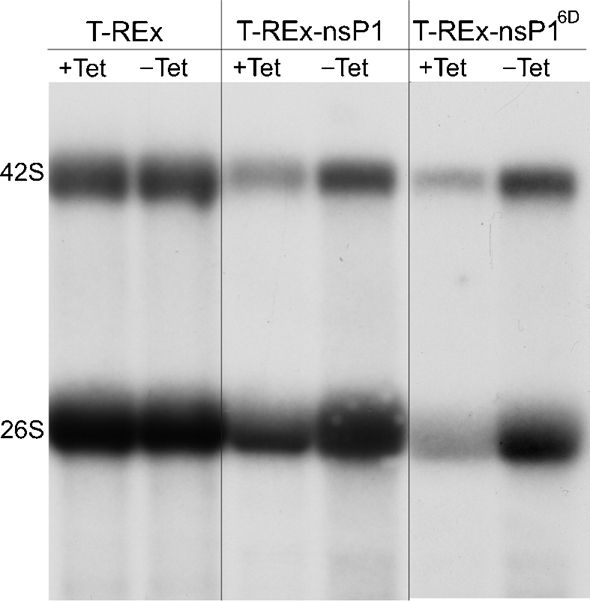
Synthesis of positive-strand viral RNAs in induced and mock-induced HEK293 T-REx, T-REx-nsP1 and T-REx-nsP1^6D^ cells. Cells grown on 35 mm plates were induced with tetracycline or mock induced and after 12 h were infected with SFV4(3H)-Rluc at an m.o.i. of 0.6. Total RNA from cells was extracted at 12 h p.i. and 5 μg of each sample was used for Northern blotting. Viral RNAs were probed with labelled RNA complementary to the positive-strand of SFV RNAs. +Tet, Tetracycline-induced cells; –Tet, uninduced controls. 42S and 26S indicate the genomic and sgRNAs of SFV4(3H)-Rluc, respectively.

Rluc activity produced by SFV4(3H)-Rluc or SFV4-StRluc depends on copy number of viral RNAs, availability of these for translation, rates of translation, extent and timing of shut-off of host cell and replicase translation, and stability of the marker protein. To determine whether nsP1 or nsP1^6D^ expression affected levels of viral RNA, total RNA was extracted from induced and mock-induced HEK293 T-REx, T-REx-nsP1 and T-REx-nsP1^6D^ cells 12 h after infection with SFV4(3H)-Rluc and analysed by Northern blotting. The amounts of viral RNAs in mock-induced HEK293 T-REx and T-REx-nsP1 cells were similar to each other, whilst the viral RNA level in uninduced T-REx-nsP1^6D^ cells was slightly lower (Fig. 6). Treatment of HEK293 T-REx cells with tetracycline had no effect on viral RNA levels. Induction of nsP1 expression decreased the amount of genomic RNA and, to some extent, the amount of sgRNA (Fig. 6). Thus, nsP1 expression specifically inhibited the synthesis of genomic RNA. In contrast, induction of nsP1^6D^ expression resulted in a strong reduction of both genomic and sgRNAs (Fig. 6). The results obtained by Northern blot analysis and those obtained by analysis of marker protein expression from recombinant viruses correlated and indicate that expression of nsP1 and nsP1^6D^ exerts different effects on SFV replication.

## DISCUSSION

Inducible expression of recombinant protein from stable cell lines has several advantages over systems based on transient expression. Firstly, induction with tetracycline causes no damage to the cells, reaches efficiencies close to 100 % and, on its own, does not affect SFV infection. Secondly, the level of recombinant gene expression is precisely controlled, as all cells in any selected cell clone have a fixed copy number of recombinant genes. Importantly, inducible systems allow expression of potentially toxic viral proteins, and inducible cell lines do not adapt to constant expression of the recombinant protein. For these reasons, stable inducible cell lines represent excellent tools for analysis of the *in vivo* properties of individual virus proteins and for studies of the effects of these on cellular metabolism, viability and virus replication. That nsP1 is a stable protein with a half-life of over 4 h, both in SFV-infected and tetracycline-induced cells, suggests that it may act in replicase complexes to stabilize more unstable viral proteins such as nsP4. It was previously found that infection of stable Hela-(SV) nsP1 cells with SV resulted in a rapid decrease in methyltransferase activity, which was suggested to have resulted from virus-induced shutdown of cellular transcription and translation (Li *et* al., 1997). This explanation assumed that ‘cellular’ nsP1 was rapidly degraded, although nsP1 stability was not measured. The high stability of SFV nsP1 suggests that the suppression of ‘cellular’ nsP1 activity by SV infection (Li *et al.*, 1997) is unlikely to have originated from virus-induced shutdown of cellular protein expression; it is perhaps more likely to have resulted from virus-induced rearrangement of cellular membranes or virus-induced changes in the general metabolism of the cell.

Expression of nsP1 led to dramatic morphological changes in induced cells, especially in the plasma membrane. This finding is consistent with the observation that transient expression of nsP1 alone is capable of inducing the formation of filopodia-like structures (Laakkonen *et al.*, 1998; Spuul *et al.*, 2007). The main difference in the localization of nsP1 in induced T-REx-nsP1 cells compared with infected cells was the lack of virus-induced intracellular replication complexes. Again, these data are coherent with the observation that, on its own, nsP1 is unable to induce formation of replication complex-like structures (Salonen *et al.*, 2003). Surprisingly, these morphological changes did not affect cell metabolism or viability. Indirectly, these findings support the hypothesis that nsP2 is the main factor responsible for the shutdown of cellular transcription/translation and induction of cell death in SFV-infected cells (Garmashova *et al.*, 2007). That expression of nsP1 or nsP1^6D^ did not reduce the number of cells in which SFV initiated replication also indicates that some other non-structural protein, again most probably nsP2, is the main mediator of superinfection exclusion (Karpf *et al.*, 1997; Sawicki *et al.*, 2006).

Expression of nsP1 in HEK293 T-REx cells interfered with virus multiplication, resulting in reduced levels of virus genomic RNA, reduced levels of replicase proteins, delayed synthesis of structural proteins and reduced levels of infectious virus. SFV infection of induced T-REx-nsP1 cells closely resembled the Arg183 mutant of SV, which, due to a defect in negative-strand RNA synthesis, generates fewer replicase complexes but has enhanced sgRNA synthesis (Fata *et al.*, 2002). It can be speculated that nsP1 expression results in lower numbers of virus replicase complexes due to competition with P123 for binding of nsP4 (Shirako *et al.*, 2000; Salonen *et al.*, 2003), incorrect incorporation into replicase complexes, inhibition of P123 or P1234 binding to membranes or interference with the translocation of replicase complexes. A reduction in replicase complex formation would result in reduced RNA replication, reduced levels of genomic RNA and reduced levels of replicase proteins, as was observed. The delayed accumulation of structural proteins could also be explained by a reduction in the number of virus replication complexes. Fewer replication complexes would result in a delay in the early synthesis of subgenomic transcripts and structural polyproteins. However, with time, perhaps these could, as was observed, accumulate to the levels seen in control cells (no nsP1 expression) due to exhaustion of metabolites or saturation of synthesis systems. An alternative explanation for the accumulation to ‘normal’ levels by 12 h of the subgenomic transcripts and structural polyproteins would be that nsP1 expression actively promotes increased production of sgRNA. Whatever the explanation, in nsP1-expressing cells, sufficient structural proteins were expressed and genomic RNA packaged to result in relatively high yields of virions.

In Hela cells, stable expression of mutated forms of nsP1 of SV(LM21) interferes with SV infection (Li *et* al., 1997). Similarly, the inhibition of SFV multiplication by nsP1^6D^ was also significant, but was different to nsP1. The extent of inhibition was far less than observed with the terminal deletion mutants of SV(LM21). The more prominent inhibitory effects of SV nsP1 expression observed by Li *et al.* (1997) may be the consequence of their much higher recombinant protein expression levels. The inhibitory effect of nsP1^6D^ was relatively mild at early stages of infection but increased over time. That the effects of expression of nsP1 and nsP1^6D^ on SFV infection were different suggests that the mechanism(s) through which these proteins affect SFV infection may be different. Indeed, several differences in biological properties of nsP1 and nsP1^6D^ have been revealed. nsP1^6D^ cannot be palmitoylated, is less tightly bound to cellular membranes and a significant fraction of it is localized in the cytoplasm of transfected cells (Ahola *et* al., 2000; Žusinaite *et* al., 2007). SFV nsP1^6D^ is also unable to interact with nsP4 (Žusinaite *et al.*, 2007). It cannot therefore compete with P123 for binding of nsP4 and may therefore be less efficient than wt nsP1 at blocking early replicase complex formation, resulting in the observed lower suppression of genomic RNA synthesis and non-structural protein expression. However, location of nsP1^6D^ on membranes could still result in incorporation into replicase complexes or affect replicase membrane interactions and thereby reduce replicative efficiency. nsP1^6D^ could still compete with replicase complexes for substrates for cap synthesis, as has also been suggested for one of the truncated forms of nsP1 of SV(LM21) (Li *et al.*, 1997).
